# Comparison of modification sites in glycated crystallin in vitro and in vivo

**DOI:** 10.1007/s00216-015-8487-7

**Published:** 2015-01-31

**Authors:** Martyna Kielmas, Monika Kijewska, Alicja Kluczyk, Jolanta Oficjalska, Bożena Gołębiewska, Piotr Stefanowicz, Zbigniew Szewczuk

**Affiliations:** 1 Faculty of Chemistry, University of Wrocław, 50-137 Wrocław, Poland; 2 Lexum Kliniki Okulistyki, 53-332 Wrocław, Poland; 3 Fundacja na Rzecz Rozwoju Nauki i Medycyny, 50-315 Wrocław, Poland

**Keywords:** Crystallin, Mass spectrometry, Solid phase synthesis, Amadori products, Non-enzymatic glycation

## Abstract

Glycation of α-crystallin is responsible for age- and diabetic-related cataracts, which are the main cause of blindness worldwide. We optimized the method of identification of lysine residues prone to glycation using the combination of LC-MS, isotopic labeling, and modified synthetic peptide standards with the glycated lysine derivative (Fmoc-Lys(*i*,*i*-Fru,Boc)-OH). The in vitro glycation of bovine lens α-crystallin was conducted by optimized method with the equimolar mixture of [^12^C_6_]- and [^13^C_6_]d-glucose. The in vivo glycation was studied on human lens crystallin. The glycated protein was subjected to proteolysis and analyzed using LC-MS. The results of in vitro and in vivo glycation of α-crystallin reveal a different distribution of the modified lysine residues. More Amadori products were detected as a result of the in vitro reaction due to forced glycation conditions. The developed method allowed us to identify the glycation sites in crystallin from eye lenses obtained from patients suffering from the cataract. We identified K166 in the A chain and K166 in the B chain of α-crystallin as major glycation sites during the in vitro reaction. We found also two in vivo glycated lysine residues: K92 in the B chain and K166 in the A chain, which are known as locations for Amadori products. These modification sites were confirmed by the LC-MS experiment using two synthetic standards. This study demonstrates the applicability of the LC-MS methods combined with the isotopic labeling and synthetic peptide standards for analysis of post-translational modifications in the biological material.

**Electronic supplementary material:**

The online version of this article (doi:10.1007/s00216-015-8487-7) contains supplementary material, which is available to authorized users.

## Introduction

Alpha-crystallin is a dominant protein of the mammalian eye lenses, essential for the maintenance of the transparency and its refractive properties [1]. It consists of two related subunits: αA and αB, with about 55 % sequence similarity [2]. These subunits occur mainly as a heterogeneous complex within mass range of approximately 600–800 kDa (known as a polydisperse oligomer). The αA subunit is specific for lenses, whereas the αB subunit appears also in other tissues, including the retina, heart, brain, lung, and skeletal muscle [3]. Moreover, increased levels of αB-crystallin are typical for many neurodegenerative disorders, diabetic conditions, and tumors [4].

The α-crystallin belongs to a small heat-shock proteins (sHsp) family of intracellular molecular chaperone proteins [5]. As a molecular chaperone, it protects other lens proteins from the effects of UV irradiation, high temperature, and chemical compounds, preventing the aggregation and denaturation of target proteins [6]. Indeed, it binds to early, partially unfolded intermediate states of other proteins by hydrophobic binding sites [7]. This reversible interaction reduces the formation of aggregation-prone late unfolding intermediates [8, 9]. It has been found that two peptides within αA- and αB-crystallin act as molecular chaperones, similar to the native molecules. The smallest peptide fragments that display a maximal antiaggregation activity are [70–88] in αA-crystallin (mini-αA-crystallin) and [73–92] in αB-crystallin (mini-αB-crystallin) [10].

Due to the fact that the lens crystallins are long-lived proteins, they undergo various post-translational modifications including oxidation, glycation, isomerization, deamidation, racemization, and truncation. These modifications are the major factors which promote protein aggregation and may contribute to progression of lens opacification. Among these modifications, the non-enzymatic reaction of α-crystallin with reducing sugars has been reported to be one of the most significant factors leading to the age-related cataract, in particular for diabetic patients [11–13]. This reaction, known as glycation or Maillard reaction (see Electronic Supplementary Material (ESM) Fig. S1), involves free amino groups in proteins (lysine or the N-terminal amino acid) and the aldehyde group of the aldose sugar, in that case glucose. The first stable product is the ketoamine which is also called the Amadori product. This product finally transforms into stable modifications on proteins that are known as advanced glycation end products (AGEs). The presence of AGEs leads to conformational changes and alterations of the protein-protein and protein-water interactions, affecting the lens protein crosslinking and aggregation, and, in consequence, the transparency of the lens [14].

Lysine residues are known to be the major glycation sites in proteins. Human, bovine, and rat αA-crystallin and αB-crystallin contain 7 and 10 lysine residues, respectively. Although all the lysine residues are potential glycation sites, studies have shown regioselectivity in the glycation of various lysine residues [15, 16].

In the first report, where glycation sites of α-crystallin have been detected, Abraham et al. studied in vitro glycation of the calf lens [15]. Two different approaches were applied to determine the sites of the reaction. In the first approach, α-crystallin was labeled by ^14^C glucose, followed by digestion by chymotrypsin. The resulting peptides were purified by RP HPLC. In the second approach, α-crystallin was modified by non-labeled glucose and after proteolysis glycated peptides were subjected to phenylboronate affinity chromatography and RP HPLC. The major reaction sites were identified by FAB-MS as K11 and K78 in αA-crystallin and K90 and/or K92 in αB-crystallin.

The second study, also presented by Abraham et al. [17], concerned a connection between mutation in the glycation site, i.e., a change from the lysine to the threonine residue, and the level of protein glycation after in vitro reaction with 100 mM fructose. The experiment confirmed that K11 is the major glycation site in αA-crystallin because there was a 33 % decrease in glycation after K11T mutation, whereas the mutation of K78 (K78T) led to only a 17 % decrease in glycation level. K166 also seems to be an important glycation site because a 27 % decrease in glycation was observed in K166T. The residues K11, K78, and K166 in αA-crystallin (K78 being the least reactive of the three) and K90, K92, and K166 in αB-crystallin were indicated to be the major glycation sites when ascorbic acid was used as a glycating agent [16].

The research concerning glycation in vivo of α-crystallin [18] was conducted on urea-soluble lens protein fractions from the cataract lenses of 1-month-old Sprague-Dawley rats. This material was treated with 1 M [^3^H]NaBH_4_. Isolated high molecular weight (HMW) aggregates were separated by molecular sieve HPLC method. After reduction with β-mercaptoethanol, they were subjected to polyacrylamide gel electrophoresis and proteolysis with chymotrypsin and trypsin. The glycated peptides were enriched by boronate affinity chromatography, purified by RP HPLC, and the fractions were examined for radioactivity. These studies indicated that K11, K78, and K166 in αA-crystallin and K166 in αB-crystallin were the most frequently modified residues in the αA- and αB-crystallin chains.

In this report, we attempted to identify the lysine residues which are the most susceptible to glycation during the model in vitro studies, as well as in vivo in patients suffering from cataract. Diabetes is a major risk factor for this disease, leading ultimately to the loss of vision [19–21]. The identification of modified residues is crucial in studies of the post-translational modifications (PTM) in α-crystallin for the future recognition of the mechanism of diabetic complications combined with cataract changes. For this purpose, the whole trypsin hydrolysate of the biological material from human lenses was analyzed using LC-MS. We also synthesized glycated standards, using a method developed by us, based on Fmoc-Lys(*i*,*i*-Fru,Boc)-OH [22]. A mixture of the standards and the obtained hydrolysate of lenses were subjected to the LC-MS analysis. These results were compared with the data obtained after in vitro glycation using the mixture of [^12^C_6_]- and [^13^C_6_]-isotopically labeled d-glucose, which was applied at high, nonphysiological concentrations. This approach was previously developed by us for detection of early [23–25] as well as advanced glycation sites [26].

The advantage of the presented method is the fast, fully tested procedure for confirmation of detected compound in biological samples. It is worth noting that preparation of the biological material has been also significantly simplified.

## Materials and methods

### Reagents

Reagents including α-crystallin isolated from bovine eye lens, isotopically labeled [^13^C_6_]d-glucose, 99 % ^13^C, [^12^C_6_]d-glucose, *O*-(6-chlorobenzotriazol-1-yl)-*N*,*N*,*N*′,*N*′-tetramethyluronium tetrafluoroborate (TCTU), dithiothreitol (DTT), ammonium bicarbonate (NH_4_HCO_3_), trifluoroacetic acid (TFA), triisopropylsilane (TIS), and solvents: *N*,*N*-dimethylformamide (DMF), dichloromethane (DCM), methanol (MeOH), and acetonitrile of the LC-MS grade were purchased from Sigma-Aldrich and were used without further purification.

Trypsin (from bovine pancreas, TPCK treated, Sigma-Aldrich) was dissolved in purified water at the concentration of 1 mg/1 ml and used for the described experiments. The Sep-Pak C18 Plus Light Cartridges (Waters) were used for desalting (parameters: 130 mg sorbent per cartridge, 55–105 μm particle size, 50/pk). For dialysis, a Pierce SnakeSkin Pleated Dialysis Tubing was used (Thermo Scientific, 10 K MWCO). Formic acid at the concentration of 99 % was purchased from Merck.

Biological samples of lens crystallin proteins obtained from the human patients suffering from diabetes were supplied by the eye clinic Lexum (Wroclaw, Poland). The fragmented lens particles are a waste material after the regular (routine) cataract surgery. Obtained materials were intended for disposal. Moreover, the fragmented portions of lens originating from different patients were collected in one dialysis cassette so the presented results cannot be assigned to a specific patient. The cataract operation is conducted in the operating room at the aseptic conditions. The operation consists in grinding patient`s eye lens with ultrasounds. During the operation, the Ringer liquid is being consistently infused. The fragmented lens particles are aspirated to disposable, sterile cases filled with liquid. Cases and drains used for the operation are an integrated circuit that is not contaminated with any biological material. Samples of crystallin were delivered in physiological saline solution at the NaCl concentration of 0.9 %.

An amino acids sequence of chains of bovine and human alpha-crystallin was in accordance with the UniProt Knowledgebase (UniProtKB).

### Experiment A (*glycation* in vitro)

Sample of α-crystallin was glycated according to the optimized method published previously [23, 26, 27] using an equimolar mixture of [^12^C_6_]d-glucose and [^13^C_6_]d-glucose. Samples were mixed with these sugars and dissolved in water to give a protein to sugar molar ratio of 1:1000 (experiments A1–A4). Results of additional experiments with the use a protein to sugar molar ratio 1:500 (experiments A5, A6), and 1:370 (experiments A7, A8) are presented in Online Recourse. The samples were lyophilized. To achieve the glycation, the dry lyophilisate was heated at 80 °C for 25 min.

#### Dialysis

Samples were dissolved and dialyzed using a SnakeSkin Dialysis Tubing for 24 h.

#### Reduction

The glycated protein (0.5 mg) was dissolved in 50 mM NH_4_HCO_3_ buffer solution. A solution of 200 μl DTT (200 mM) was added and then the mixture was incubated at 60° for 30 min.

#### Proteolysis

The trypsin solution was added to the mixture of the reduced protein (0.5 mg) to obtain the 1:10 (A1–A3 and A5–A8) or alternatively 1:1 (A4) enzyme:substrate mass ratio. The mixture was incubated at 37 °C for 24 h. Digestion was terminated by the addition of 10 μl of formic acid. The samples were subjected to LC-MS analysis.

### Experiment B (glycation in vivo)

**Fig. 1:**
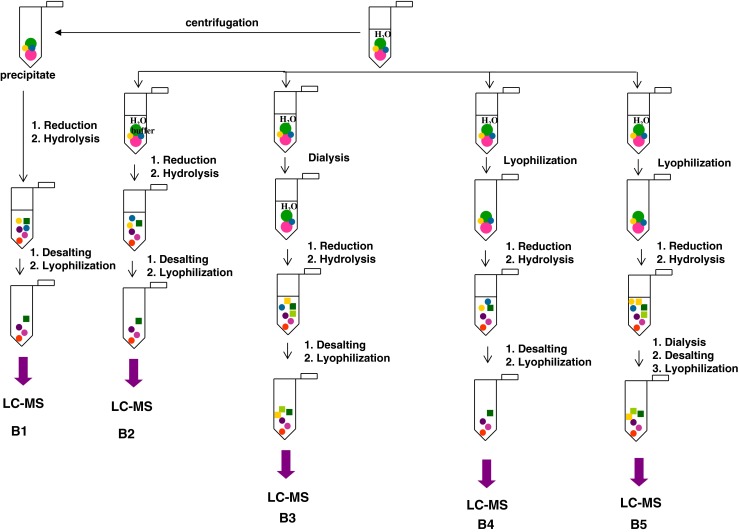
Diagram of the procedures for preparation of the biological material for LC-MS analysis (B1–B5)

The sample B, which contained the biological material obtained from 11 human lenses in the volume of 600 ml of 0.9 % saline solution, was first centrifuged for 20 min (14,000 RPM, 16,873×*g*) to separate the insoluble precipitate from the supernatant (see Fig. 1, B1). Four 100-ml portions of the supernatant were taken for further analysis. Four different strategies were applied to compare the obtained fragments. The experiments consisted of the same steps which were performed in different order. The amounts of reagents (trypsin and dithiothreitol) were calculated on the basis of the amount of proteins isolated from one lens according to the literature [28].

#### Reduction

The lyophilized samples were dissolved in 50 mM NH_4_HCO_3_ buffer solution (3 ml). The solution of DTT (45 μl, 45 mM, in water) was added and then the mixture was incubated at 50 °C for 15 min.

#### Proteolysis

The trypsin solution was added to the mixture of the reduced proteins to obtain the 1:10 enzyme:substrate mass ratio. The mixture was incubated at 37 °C for 24 h. Digestion was terminated by the addition of 10 μl of formic acid.

#### Desalting

The samples (after enzymatic hydrolysis) were lyophilized and then desalted on an RP-C18 cartridge (Sep-Pak). The hydrolysates were eluted by 60 % acetonitrile in water. The sample after lyophilization was subjected to the LC-MS analysis.

The sample B2 was treated in the same way as the precipitate (B1). In the case of B3, the procedure was preceded by dialysis whereas two samples (B4–B5) were first lyophilized. All the samples were subjected to reduction by DTT (100 μl, 45 mM, in water), hydrolysis, and desalting on an RP cartridge, and analyzed by LC-MS.

The procedures for preparation of two additional portion of biological materials (experiments: C and D) obtained from the patients suffering from cataract after cataract surgery are presented in ESM (Fig. S3 and S4).

### Synthesis of glycated peptides

The synthesis of two glycated peptides of glycated human α-crystallin (VK(Fru)VLGDVIEVHGK and EEK(Fru)PTSAPSS) was performed according to the Fmoc protocol using the Wang resin. The sequences of these peptides were [91–103] from the αB chain and [164–173] from the αA chain. The synthesis was conducted using synthetic protected fructolysine Fmoc-Lys(*i*,*i*-Fru,Boc)-OH (ESM Fig. S2) obtained by procedure described previously by Stefanowicz et al. [23]. TCTU was used as a coupling reagent. The peptides were cleaved from the resin using TFA/water/TIS (90:5:5, *v*/*v*) for 8 h at a room temperature and precipitated with cold diethyl ether.

### LC-MS analyses of biological material and synthetic peptides

The first LC-MS experiment was conducted for the synthetic peptides. Then the trypsin hydrolysate of human lens crystallin from the B3 sample was analyzed. Finally, this hydrolysate was mixed with two synthetic peptides for a third LC-MS experiment.

### LC-MS measurements

The LC-MS analyses were performed in the Laboratory of Mass Spectrometry at the Faculty of Chemistry, University of Wroclaw using an Agilent 1200 HPLC system coupled to a micrOTOF-Q mass spectrometer (Bruker Daltonics, Germany). The micrOTOF-Q instrument equipped with an ESI source with an ion funnel was operated in the positive ion mode and calibrated before each analysis with the Tunemix™ mixture (Bruker Daltonics, Germany) in a quadratic method. Argon was used as a collision gas. For separation, an Aeris PEPTIDE, Phenomenex (50 × 2.1 mm, 3.6 μm) column was used with elution gradient of 0–100 % B in A (A = 0.1 % HCOOH in water; B = 0.1 % HCOOH in acetonitrile) over 62 min (flow rate 0.05 ml/min, room temperature). In LC-MS/MS experiments, the collision energy eV was selected after CID studies on the synthetic model peptides.

### MS/MS analysis

The FT-ICR instrument (Bruker Daltonics, Germany) equipped with an ESI source with an ion funnel was operated in the positive ion mode and calibrated before each analysis with the Tunemix™ mixture (Bruker Daltonics, Germany) in a quadratic method. Argon was used as a collision gas. In MS/MS experiments, the collision energy (15 eV) was optimized for the best fragmentation.

### Data analysis

The mass list generated by a DataAnalysis program, 4.0 (Bruker, Germany) was analyzed using a home-developed software written in JAVA. The procedure is based on searching for pairs of ions (light and heavy forms of the Amadori product) of equal abundances in which the difference of monoisotopic mass equals 1.003 × *n*, where *n* is the number of carbon atoms derived from glucose. The accepted error of mass difference was below 0.02 Da, while the accepted difference of abundances was below 10 %. The program calculates the theoretical masses of peptides obtained from in silico digestion taking into consideration the defined specificity of trypsin. Then it assigns the peptide sequences to peaks from the scans of the LC-MS data set and generates lists of potential glycated peptides using mass shifts characteristic for early glycation products.

## Results and discussion

The products of reaction of α-crystallin with d-glucose were analyzed for both types of glycation: in vitro and in vivo. The results of the in vivo glycation (experiments: B, C, D) were compared with the data obtained from the in vitro glycation using high concentrations of isotopically labeled sugar (experiments: A1–A8) and in the last stage confirmed by the synthetic glycated standards.

### Study on in vitro glycation of α-crystallin (experiments A1–A8)

The first stage of our study was the in vitro glycation of commercially available crystallin from bovine eye lens with [^13^C_6_]d-glucose (^13^C_6_ Glc). It was conducted to create a reference data set for glycation in vivo required to compare the process of glycation at the physiological and forced conditions. The samples of α-crystallin were mixed with an equimolar mixture of [^12^C_6_]d-glucose and [^13^C_6_]d-glucose at the different molar protein to glucose ratios: 1:370 (A7–A8), 1:500 (A5–A6), and 1:1000 (A1–A4). The glycation was conducted by the optimized procedure published by us [26, 27]. The samples were dialysed, reduced, and hydrolysed by trypsin. The hydrolysates were subjected to LC-MS experiments. The obtained data sets were analyzed by a program written in Java. The identification of the glycated peptides was based on a characteristic isotopic distribution. The isotope labeling leads to a characteristic signal distribution in mass spectra—the doublet pattern of glycated peptides with the equal intensity and a mass difference of 6.018 Da, which simplifies the interpretation of the LC-MS results.

**Fig. 2:**
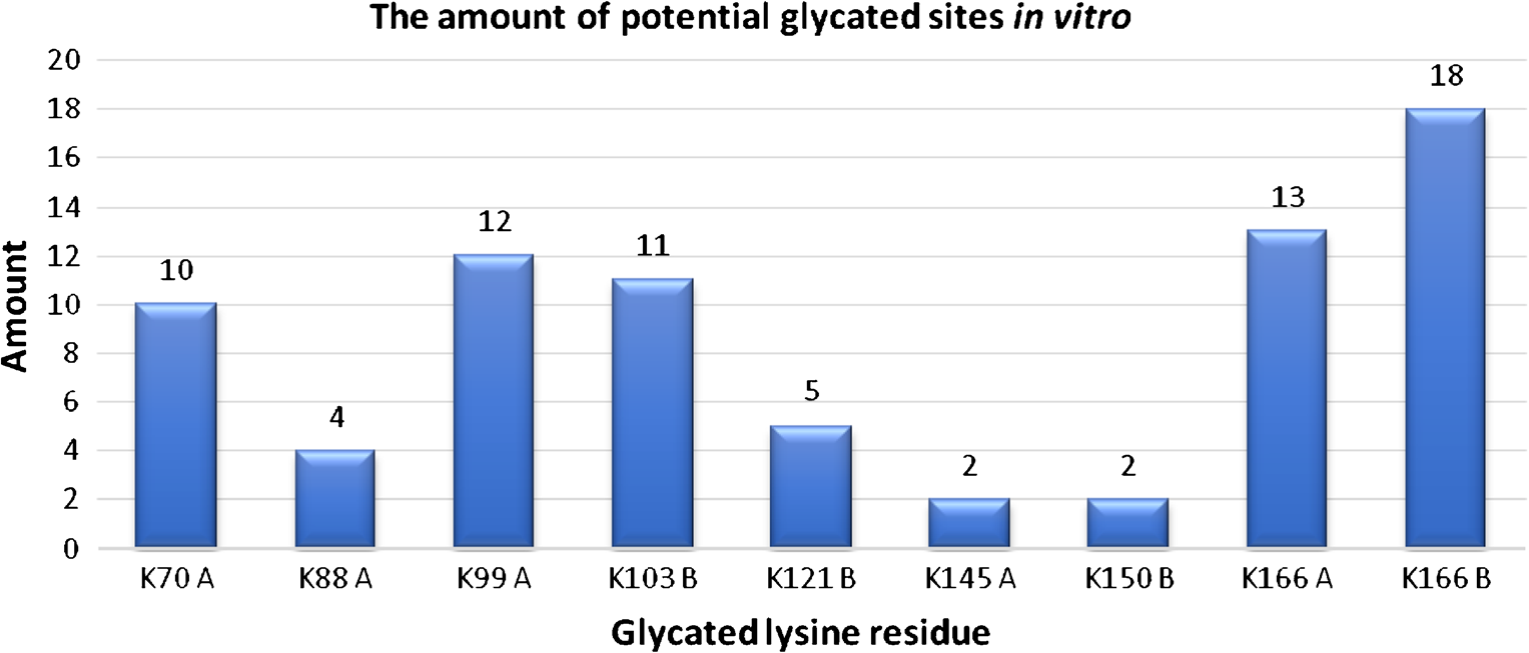
The scheme of glycated lysine residues in αA- and αB-crystallin on the basis of occurrence of the monoglycated peptides with modification in data set from in vitro experiment

The number of identified glycation sites (Table 1 and ESM Tables S1 to S7) depends on the protein to glucose ratio. In the in vitro data set for monoglycated peptides (containing one lysine residue), K166 from the αA chain and K166 from the αB chain occur the most frequently (Fig. 2). The occurrence is based on the number of detected peptide fragments in eight conducted experiments (A1–A8). Other lysine residues which are located in the αA chain: K70, K88, K99, and K145 were less frequently glycated. For the αB chain, the lysine residues K103, K121, and K150 are also identified as potential glycation sites. Taking into account the concentration of d-glucose, we observed that in fragments with one potential glycation site, K70 and K99 from the αA chain and K103 and K166 from the αB chain dominate for samples A7 and A8 (ESM Tables S6 and S7). In the case of samples A1–A6, the most prevalent sites are: K70, K99, and K166 from the αA chain and K166 from the αB chain (Table 1 and ESM Tables S3 to S7).

**Table 1:** The glycated fragments identified in experiment A1 (Materials and methods)

Observed *m*/*z*	*z*	Found MW	Calc. MW	RT^a^ [min]	Sequence	AA
601.9697	3	1802.8856	1802.8898	18–19	[158–173] α-crystallin A	K166
626.3465	5	3126.6934	3126.6772	18–19	[150–175] α-crystallin B^a^	K150, K166, K174
704.7314	3	2111.1707	2111.1838	18–19	[158–175] α-crystallin B	K166, K174
1137.6215	2	2273.2273	2273.2366	18–19	[158–175] α-crystallin B^a^	K166, K174
496.7779	4	1983.0803	1983.0888	19–20	[158–174] α-crystallin B	K166
568.3147	5	2836.5344	2836.5294	19–20	[150–174] α-crystallin B	K150, K166
662.0344	3	1983.0797	1983.0888	19–20	[158–174] α-crystallin B	K166
704.7335	3	2111.1770	2111.1838	19–20	[158–175] α-crystallin B	K166, K174
710.1376	4	2836.5191	2836.5294	19–20	[150–174] α-crystallin B	K150, K166
902.4492	2	1802.8827	1802.8898	19–20	[158–173] α-crystallin A	K166
992.5451	2	1983.0745	1983.0888	19–20	[158–174] α-crystallin B	K166
614.8909	5	3069.4154	3069.4177	20–21	[89–112] α-crystallin A	K99
662.0318	3	1983.0719	1983.0888	20–21	[158–174] α-crystallin B	K166
688.3538	2	1374.6919	1374.7031	20–21	[83–92] α-crystallin B	K90
590.4948	5	2947.4349	2947.4424	21 –22	[93–116] α-crystallin B	K103
635.9284	5	3174.6029	3174.6058	21–22	[91–116] α-crystallin B	K103
728.7490	5	3638.7059	3638.6887	21–22	[89–116] α-crystallin A	K99
704.3482	5	3516.7019	3516.7134	22–23	[93–120] α-crystallin B	K103
575.6418	3	1723.9019	1723.8992	23–24	[70–82] α-crystallin B	K72
691.3567	6	4142.0932	4142.0821	25–26	[83–116] α-crystallin B	K90, K92, K103
786.2351	6	4711.3636	4711.3532	25–26	[83–120] α-crystallin B	K90, K92, K103
788.8895	4	3151.5267	3151.5211	26–27	[79–103] α-crystallin A	K88, K99
845.6076	5	4222.9989	4222.9944	26–27	[79–112] α-crystallin A	K88, K99
959.4628	5	4792.2749	4792.2655	26–27	[79–116] α-crystallin A	K88, K99
1051.5157	3	3151.5236	3151.5211	26–27	[79–103] α-crystallin A	K88, K99
1056.7610	4	4223.0127	4222.9944	26–27	[79–112] α-crystallin A	K88, K99
581.9815	3	1742.9210	1742.9091	27–28	[66–78] α-crystallin A	K70
1308.6524	2	2615.2891	2615.2755	28–29	[79–99] α-crystallin A	K88
967.5204	3	2899.5377	2899.5179	30–31	[55–78] α-crystallin A	K70

*AA* potential glycated lysine residues

^a^Diglycated peptides. Retention time range used for averaging mass spectra [min]. Details of LC-MS are described in “Materials and methods”

According to the trypsin specificity, all the fragments could contain only one unmodified lysine residue, however, we observed several peptide fragments resulting from incomplete proteolysis. In this case, the increase in glucose concentration led to a significant limitation of the number of potential cleavage sites (K and R) due to the fact that the Amadori product on lysine residue inhibits the trypsin action.

In the case of K88 and K99, we can distinguish fragments with K99 glycated, however, there are multiple fragments containing both these lysine residues with only one glycation site.

Table 1 presents results from experiment A1 for glycation occurring at the protein to d-glucose 1:1000 molar ratio. The obtained results revealed the six most frequently glycated lysines. Three of them, K70, K88/K99, and K166 are located in the αA chain sequences [66–78]/[55–78], [79–103]/[79–112]/[79–116], and [158–173], respectively. Three others are: K90/K92/K103, K103, and K150/K166/K174 in the fragments of the sequence [83–116]/[83–120], [91–116]/[93–116]/[93–120], and [150–175]/[158–174]/[158–175] in the αB chain.

The characteristic isotopic distribution for pair of signals from the [^12^C_6_]- and [^13^C_6_]-Amadori products, their identical retention times, and the identical intensities of extracted ion chromatograms (XIC) were very strict criteria for examination of the glycated compounds. The reproducibility of our experimental results is very high which may be related to the presence of only one pure protein in the reaction mixture facilitating the interpretation of the LC-MS data.

The main outcomes of these experiments are:

- The LC-MS method was combined with a stable isotope labeling showed the regioselectivity of glycation during the in vitro conditions. Redistribution of modified lysine residues depends on concentration of d-glucose.
- We identified fragments of glycated peptides corresponding to the sites of α-crystallin most easily modified during presented model experiments with the using d-glucose at high, nonphysiological concentrations.
- In the highest concentration of d-glucose six lysine residues occur generally: K70, K88/K99, and K166 (αA chain); K90/K92/K103, K103, and K150/K166/K174 (αB chain).

### Study on in vivo glycation of α-crystallin (experiments B–D)

In the second stage of the project, we attempted to identify the lysine residues in both αA- and αB-crystallin that easily undergo glycation in lenses from the patients suffering from cataract. The biological material originating from different patients has been provided in a collective package so we could not relate the degree of modification of αA- and αB-crystallin to the age and severity of diabetes. The biological material was delivered in three portions containing 1, 2, and 11 lenses. The mixture of proteins from human lenses was treated by different protocols described in “Materials and methods” (see also in Online Resource for more details for experiments C and D). All the protocols include three main steps: dialysis/desalting, reduction, and enzymatic hydrolysis using trypsin, conducted in different order. The largest number of experiments was carried out for the sample (B) because it contained the biological material obtained from 11 lenses. The amount of reagents for reduction of the disulfide bridges and hydrolysis was calculated in relation to the proteins content in one lens according to the literature [28]. The preparation of the biological material was optimized to obtain the largest number of the modified peptides. The insoluble part of the biological material (precipitate) was separated by centrifugation and then subjected to a separate analysis. The supernatant was divided and analyzed according to the protocol presented in Fig. 1. Because of a high concentration of salt, two of the samples were subjected to dialysis at different stages of the isolation procedure. In these five experiments, each of the two identified peptide fragments was found in at least three analyses (Table 2). The retention times of three modified fragments found were identical with the results acquired from the in vitro glycation analysis. Due to the very strict criteria for examination of glycated compounds, the extracted ion chromatogram was created to prove that the retention times are the same. To confirm the authenticity of the other unmodified fragments, MS/MS analyses for selected fragments [55–65] αA chain, [146–157] αA chain, and [57–69] αB chain were carried out and the results were in agreement with the predicted fragmentation patterns. The fragmentation spectra confirmed the sequences of these fragments. The representative MS/MS spectra for three selected fragments of αA- and αB- crystallin are presented in Figs. S7, S8, S9 in the ESM.

**Table 2:** The list of identified glycated peptides in three samples (B, C, D) of the biological material (Materials and methods)

Sample	Observed *M*/*Z*	*z*	Found Mw	Calc. MW	RT^a^ [min]	Sequence	AA
B1	597.7781	2	1193.5405	1193.5299	17–18	[164–173] α-crystallin A chain	K166
B2	597.7771	2	1193.5385	1193.5299	20–21	[164–173] α-crystallin A chain	K166
	777.9455	2	1553.8753	1553.8665	23–24	[91–103] α-crystallin B chain	K92
B3	777.9481	2	1553.8805	1553.8665	23–24	[91–103] α-crystallin B chain	K92
B4	398.8547	3	1193.5406	1193.5299	20–24	[164–173] α-crystallin A chain	K166
	777.9336	2	1553.8515	1553.8665	23–24	[91–103] α-crystallin B chain	K92
B5	398.8537	3	1193.5376	1193.5299	22–23	[164–173] α-crystallin A chain	K166
C2	1194.5463	1	1193.5385	1193.5299	19–20	[164–173] α-crystallin A chain	K166
	777.9443	2	1553.8729	1553.8665	23–24	[91–103] α-crystallin B chain	K92
	785.3809	4	3137.4923	3137.5054	29–30	[79–103] α-crystallin A chain	K88, K99
D1	777.9354	2	1553.8551	1553.8665	23–24	[91–103] α-crystallin B chain	K92
D2	1194.5477	1	1193.5399	1193.5299	20–21	[164–173] α-crystallin A chain	K166

*AA* potential glycated lysine residues

^a^Retention time range used for averaging mass spectra [min]. Details of LC-MS are described in “Materials and methods”

For sample C, the procedure C1 based on first isolating of proteins using RP cartridge Sep-Pak and then reduction and hydrolysis did not provide as many fragments of the modified peptides as the procedure C2. We observed a significant loss of fragments due to the irreversible absorption on the stationary phase. The LC-MS analysis of desalted samples C1 showed a low intensity of signals corresponding to the proteins. The MS spectrum of αB crystallin is presented in ESM Fig. S5. Three modified fragments (Table 2) were found in the LC-MS analysis of the samples prepared by a second method (C2). The representative chromatogram obtained for sample C2 is presented in ESM Fig. S6. The most abundant signal corresponds to [91–103] αB-crystallin, but the intensity of the glycated peptide signals was not sufficient to perform fragmentation experiments.

Sample D consisting of two lenses was divided into three different portions. The analyses of samples D1 and D2, which contained the soluble and insoluble parts of the biological material, respectively, were performed according to the same procedure as described for sample C2. In contrast, the first step of lyophilization was omitted for sample D2. The sample was adjusted to pH 8 using 50 mM NH_4_HCO_3_ buffer solution and then reduction and hydrolysis were carried out. The obtained results are presented in Table 2. In this experiment, only one modified fragment [91–103] from αB-crystallin was found in sample D1. The results acquired from studies on two lenses provided less information on the modified fragments, which could be correlated with the severity of diabetes and the age of patients.

The main outcomes of these experiments are:

- According to the results of several experiments, from the set of the available lysine residues the most frequently glycated are the ones at positions: K88/K99 and K166 in the αA chain and K92 in the αB chain.
- In the analyzed mass spectra, the intensity of signals corresponding to glycated peptide [91–103] αB chain with K92 was the highest in comparison to other modified fragments.
- The obtained results are consistent with the literature data for the in vivo [18] and in vitro glycation [15–17].

### Application of the synthetic glycated standards for analysis of the Amadori products in the biological material

The third step of our analysis was a verification of structure for the glycated peptides identified in the second stage of our research. For this purpose, the synthesis of two glycated peptides (tryptic fragments of glycated human α-crystallin) was performed. The sequences of these peptides were [91–103] of the αB chain and [164–173] of the αA chain. The synthesis was conducted according to the procedure described by us previously, using fully protected synthetic fructolysine Fmoc-Lys(*i*,*i*-Fru,Boc)-OH [22].

According to the literature [29], the sequencing of peptides with the Amadori products by collision-induced dissociation results in dissociation of the weakest bonds. The consequences are neutral losses from the hexose moiety or elimination of the whole hexose moiety before dissociation of any peptide bond. Therefore, the obtained MS spectra are complicated and difficult to interpret. The elimination of the sugar moiety from an Amadori product may also result in an incorrect identification of the modification site. Moreover, for the studied native hydrolysates, the intensity of detected signals was really low. Due to these facts, the application of MS/MS techniques for identification of the modification location was unpractical. Many overlapping MS signals with a similar *m*/*z* ratio further complicate interpretation of the data.

**Fig. 3:**
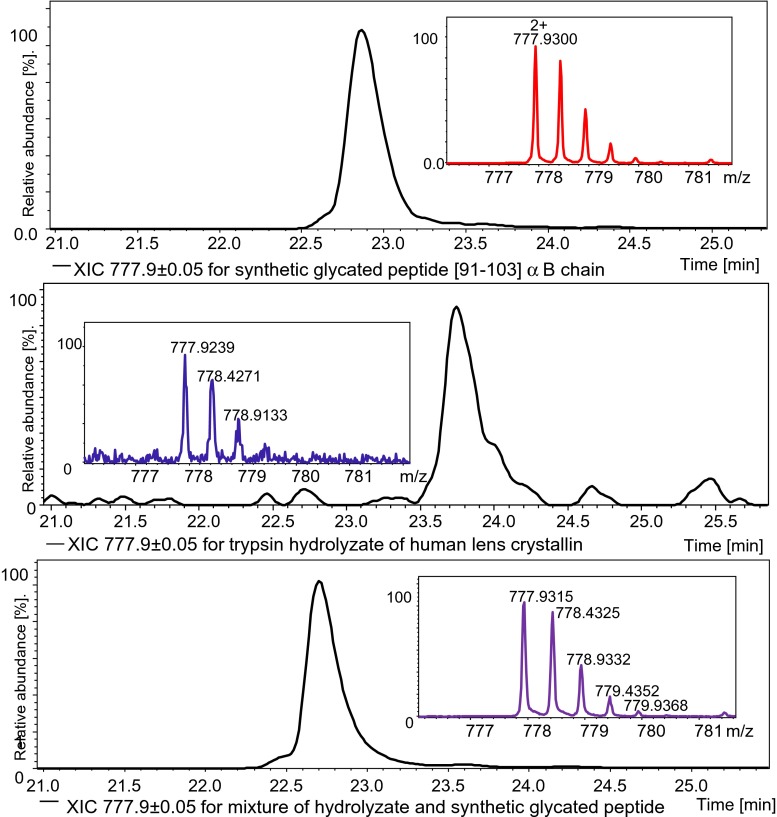
The comparison of XIC and mass spectra for three LC-MS analyses (see “Materials and methods” for details) on the glycated peptide [91–103] from the αB chain

**Fig. 4:**
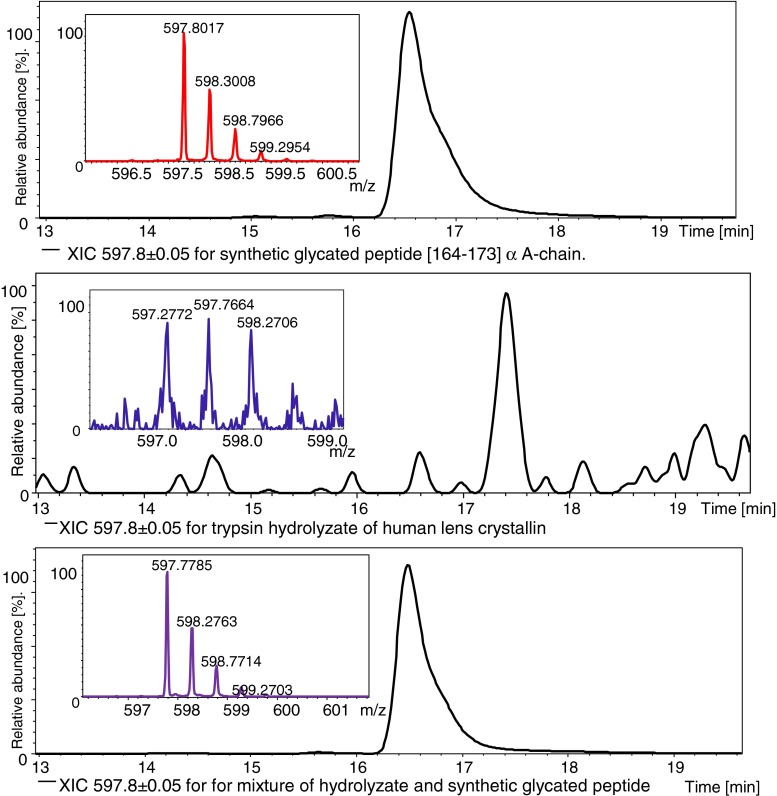
The comparison of XIC and mass spectra for three LC-MS analyses (see “Materials and methods” for details) on the glycated peptide [164–173] from the αA chain

For the above-mentioned reasons, the LC-MS method was used to confirm the authenticity of two selected glycated peptides. At first, we conducted an LC-MS experiment for the synthetic native peptide. Then the trypsin hydrolysate of human lens crystallin (B3) was analyzed using the same gradient conditions of LC-MS. Next, the hydrolysate was mixed with two synthetic native peptides for another LC-MS experiment. As a result, we detected these compounds in synthetic native forms, in hydrolysate, and the mixture with different intensities of XIC and signals in the mass spectrum. The intensities of peaks in chromatograms and mass spectra corresponding to both peptides increased in the third experiment, which is presented in Figs. 3 and 4.

It is worth noting that the retention times during LC-MS analysis of the analyzed compounds were nearly identical in comparison with the retention time obtained for the native hydrolysate. Such parameters as mass accuracy, intensities of XIC, and retention times confirmed the locations of modification sites. It also validated our results for these extremely complex mixtures of the biological material.

## Conclusions

Our results confirmed the regiospecificity of glycation at different conditions. The in vitro experiments with the use of isotope labeling and LC-MS indicated many reaction sites. The distribution of these sites is slightly different for various concentrations of [^12^C_6_]- and [^13^C_6_]d-glucose. In this data set, considered for monoglycated peptides with one reaction site, glycation at lysine residues: K166 of the αA chain and K166 of the αB chain generally occurs. These lysine residues are the most susceptible for glycation at high glucose concentrations. In the experiment conducted for the in vivo glycation, we detected a lower amount of compounds than in the first stage of our research. We confirmed the identity of two peptides with the Amadori products using the synthetic models in LC-MS analyses: [91–103] αB chain and [164–173] αA chain.

These products occurred in each performed analysis of the biological material from different patients suffering from cataract. The detected lysine residues: K92 in the αB chain and K166 in the αA chain are the most frequently glycated during the disease process. The confirmation of these glycation sites may allow for a future quantification of the detected fragments. This result is essential for understanding the modification mechanism in α-crystallin during age-related and diabetic cataract. The verification of the disease history with these data can present the effect of diabetic conditions and age-related changes on the modification of α-crystallin. Such a method may be also used for quantitative determination of advanced glycation end products. For this purpose, new isotopically labeled peptide derivatives should be prepared.

The advantages of the presented method include the reaction rate and simplicity of the analysis by using the few steps: dialysis, reduction, proteolysis, and LC-MS analysis. In the proposed methods, the enrichment of the glycated samples is not necessary. The new methods for analysis of the in vivo glycation were optimized to obtain most of the glycated fragments. All the procedures for checking the authenticity of identified glycated peptides: the synthesis of the standards and the in vitro glycation, were thoroughly tested before [26, 27, 22].

## Electronic supplementary material

Below is the link to the electronic supplementary material.

ESM 1 (PDF 172 kb)
