# Supplementary material for: Comparison of modification sites in glycated crystallin in vitro and in vivo
Source: Anal Bioanal Chem. 2015 Jan 31;407(9):2557–67. doi: 10.1007/s00216-015-8487-7 (PMC4365289; doi:10.1007/s00216-015-8487-7)
Supplement: Supplementary file 1 — (PDF 172 kb) [file 216_2015_8487_MOESM1_ESM.pdf]

Analytical and Bioanalytical Chemistry

Electronic Supplementary Material

Comparison of modification sites in glycated crystallin in vitro and in vivo

Martyna Kielmas, Monika Kijewska, Alicja Kluczyk, Jolanta Oficjalska,
Bożena Gołębiewska, Piotr Stefanowicz, Zbigniew Szewczuk

Materials and methods

Experiment C (glycation *in vivo*)

The sample, which contained the biological material obtained from one human lens in the volume of 100 ml of 0.9 % saline solution, was divided into two parts. Two parallel analyses (C1 and C2) were performed to verify the reproducibility of the results
(**Fig. S3**). The experiments consisted of the same steps which were performed in different order.

Reduction

The lyophilized samples were dissolved in 50 mM NH_4HCO_3 buffer solution (3 ml). The solution of DTT (45 µl, 45 mM, in water) was added and then the mixture was incubated at 50 ºC for 15 min.

Proteolysis

The trypsin solution was added to the mixture of the reduced proteins to obtain the 1:10 enzyme:substrate mass ratio. The mixture was incubated at 37° for 24 h. Digestion was terminated by the addition of 10 µl of formic acid.

Desalting

1. The sample of biological material (50 ml) was desalted using an RP-C18 cartridge (Sep-Pak). The mixture of proteins was eluted by 10 %, 25 %, and 70 % acetonitrile in water. The samples after lyophilization were subjected to the LC–MS analysis (C1).
2. The samples (after enzymatic hydrolysis) were lyophilized and then desalted on an RP-C18 cartridge (Sep-Pak). The hydrolyzates were eluted by 60 % acetonitrile in water. The sample after lyophilization was subjected to the LC–MS analysis (C2).

Experiment D (glycation *in vivo*)

The sample, which contained the biological material obtained from two human lenses in the volume of 300 ml of 0.9 % saline solution, was divided into aliquots of 100 ml each. Two portions contained only well soluble biological material and in the third one a precipitation was noticed. Two different strategies were performed to compare the obtained results (**Fig. S4**). In the first approach (D1**,** D3), the same procedure was carried out as for sample C, using the following amounts of reagents: DTT (60 µl, 45 mM, in water) and the enzyme at the 1:10 ratio to the protein as in experiment B1. In the second approach, the sample was subjected to reduction by DTT and then hydrolysis without lyophilization (D2). The pH of the sample solution was adjusted to 8 using 50 mM NH_4HCO_3. After enzymatic hydrolysis the sample was lyophilized and then desalted as in experiment C2. The sample after lyophilization was subjected to the LC–MS analysis.

Table S1 The glycated fragments identified in experiment A2 (Materials and Methods). AA- potential glycated lysine residues

m/z	z	exp. mass	calc. mass	time	sequence	AA
590.7618	2	1179.5079	1179.5143	15 min	[164-173] alpha crystallin A	K166
651.8390	2	1301.6623	1301.6715	17 min	[164-174] alpha crystallin B	K166
663.7217	5	3313.5694	3313.5739	26 min	[79-103] alpha crystallin A*	K88, K99
731.8480	6	4385.0410	4385.0473	26 min	[79-112] alpha crystallin A*	K88, K99
581.9762	3	1742.9051	1742.9091	27 min	[66-78] alpha crystallin A	K70
801.6717	4	3202.6555	3202.6721	30 min	[121-149] alpha crystallin B	K121

* Asterisk indicates diglycated peptides. Retention time range used for averaging mass spectra [min]. Details of LC-MS are described in Materials and Methods

Table S2 The glycated fragments identified in experiment A3 (Materials and Methods). AA- potential glycated lysine residues

m/z	z	exp. mass	calc. mass	time	sequence	AA
590.7616	2	1179.5075	1179.5143	16 min	[164-173] alpha crystallin A	K166
601.9668	3	1802.8769	1802.8898	18 min	[158-173] alpha crystallin A	K166
902.4464	2	1802.8771	1802.8898	18 min	[158-173] alpha crystallin A	K166
526.2862	4	2101.1135	2101.1015	19 min	[150-166] alpha crystallin B	K150
704.7312	3	2111.1701	2111.1838	19 min	[158-175] alpha crystallin B	K166
429.7423	4	1714.9379	1714.9214	20 min	[150-163] alpha crystallin B	K150
614.8902	5	3069.4119	3069.4177	20 min	[89-112] alpha crystallin A	K99
662.0333	3	1983.0764	1983.0888	20 min	[158-174] alpha crystallin B	K166
768.3600	4	3069.4087	3069.4177	20 min	[89-112] alpha crystallin A	K99
737.8642	4	2947.4255	2947.4424	21 min	[93-116] alpha crystallin B	K103
704.3513	5	3516.7174	3516.7134	22 min	[93-120] alpha crystallin B	K103
685.6173	7	4792.2663	4792.2655	26 min	[79-116] alpha crystallin A	K88, K99
799.7194	6	4792.2694	4792.2655	26 min	[79-116] alpha crystallin A	K88, K99
845.6056	5	4222.9889	4222.9944	26 min	[79-112] alpha crystallin A	K88, K99
581.9766	3	1742.9063	1742.9091	27 min	[66-78] alpha crystallin A	K70

Table S3 The glycated fragments identified in experiment A4 (Materials and Methods). AA- potential glycated lysine residues

m/z	z	exp. mass	calc. mass	time	sequence	AA
590.7645	2	1179.5133	1179.5143	15 min	[164-173] alpha crystallin A	K166
601.9705	3	1802.8880	1802.8898	18 min	[158-173] alpha crystallin A	K166
704.7319	3	2111.1722	2111.1838	18 min	[158-175] alpha crystallin B	K166, K174
902.4490	2	1802.8823	1802.8898	18 min	[158-173] alpha crystallin A	K166
1137.6186	2	2273.2215	2273.2366	18 min	[158-175] alpha crystallin B*	K166, K174
568.3132	5	2836.5269	2836.5294	19 min	[150-174] alpha crystallin B	K150, K166
662.0338	3	1983.0779	1983.0888	19 min	[158-174] alpha crystallin B	K166
704.7307	3	2111.1686	2111.1838	19 min	[158-175] alpha crystallin B	K166, K174
758.7500	3	2273.2265	2273.2366	19 min	[158-175] alpha crystallin B*	K166, K174
902.4468	2	1802.8779	1802.8898	19 min	[158-173] alpha crystallin A	K166
992.5442	2	1983.0727	1983.0888	19 min	[158-174] alpha crystallin B	K166
614.8898	5	3069.4099	3069.4177	20 min	[89-112] alpha crystallin A	K99
662.0331	3	1983.0758	1983.0888	20 min	[158-174] alpha crystallin B	K166
768.3591	4	3069.4051	3069.4177	20 min	[89-112] alpha crystallin A	K99
799.7166	6	4792.2526	4792.2655	25 min	[79-116] alpha crystallin A	K88, K99
581.9750	3	1742.9015	1742.9091	27 min	[66-78] alpha crystallin A	K70
1308.6367	2	2615.2577	2615.2755	28 min	[79-99] alpha crystallin A	K88
801.6719	4	3202.6563	3202.6721	30 min	[121-149] alpha crystallin B	K121
1602.3356	2	3202.6555	3202.6721	30 min	[121-149] alpha crystallin B	K121
890.1291	6	5334.7276	5334.7300	33 min	[55-99] alpha crystallin A	K70, K78, K88

* Asterisk indicates diglycated peptides. Retention time range used for averaging mass spectra [min]. Details of LC-MS are described in Materials and Methods

Table S4 The glycated fragments identified in experiment A5 (Materials and Methods). AA- potential glycated lysine residues

m/z	z	exp. mass	calc. mass	time	sequence	AA
902.4477	2	1802.8797	1802.8898	18 min	[158-173] alpha crystallin A	K166
614.8950	5	3069.4359	3069.4177	20 min	[89-112] alpha crystallin A	K99
662.0373	3	1983.0884	1983.0888	21 min	[158-174] alpha crystallin B	K166
704.8430	6	4223.0110	4222.9944	26 min	[79-112] alpha crystallin A	K88, K99
829.4042	4	3313.5855	3313.5739	27 min	[79-103] alpha crystallin A*	K88, K99
844.6414	5	4218.1679	4218.1656	28 min	[121-157] alpha crystallin B*	K121, K150
676.5268	6	4053.1138	4053.0946	31 min	[55-88] alpha crystallin A	K70

* Asterisk indicates diglycated peptides. Retention time range used for averaging mass spectra [min]. Details of LC-MS are described in Materials and Methods

Table S5 The glycated fragments identified in experiment A6 (Materials and Methods). AA- potential glycated lysine residues

m/z	z	exp. mass	calc. mass	time	sequence	AA
666.9947	3	1997.9606	1997.9443	19 min	[89-103] alpha crystallin A	K99
902.4510	2	1802.8863	1802.8898	19 min	[158-173] alpha crystallin A	K166
626.9939	3	1877.9582	1877.9483	20 min	[93-107] alpha crystallin B	K103
662.0404	3	1983.0977	1983.0888	20 min	[158-174] alpha crystallin B	K166
902.4507	2	1802.8857	1802.8898	20 min	[158-173] alpha crystallin A	K166
992.5551	2	1983.0945	1983.0888	20 min	[158-174] alpha crystallin B	K166
1053.5580	2	2105.1003	2105.1117	20 min	[91-107] alpha crystallin B	K92, K103
983.4938	3	2947.4579	2947.4424	21 min	[93-116] alpha crystallin B	K103
581.9829	3	1742.9252	1742.9091	27 min	[66-78] alpha crystallin A	K70
1308.6543	2	2615.2929	2615.2755	28 min	[79-99] alpha crystallin A	K88

Table S6 The glycated fragments identified in experiment A7 (Materials and Methods). AA- potential glycated lysine residues

m/z	z	exp. mass	calc. mass	time	sequence	AA
662.0346	3	1983.0803	1983.0888	19 min	[158-174] alpha crystallin B	K166
666.9914	3	1997.9507	1997.9443	19 min	[89-103] alpha crystallin A	K99
758.7503	3	2273.2274	2273.2366	19 min	[158-175] alpha crystallin B*	K166, K174
614.8904	5	3069.4129	3069.4177	20 min	[89-112] alpha crystallin A	K99
662.0364	3	1983.0857	1983.0888	20 min	[158-174] alpha crystallin B	K166
590.4973	5	2947.4474	2947.4424	21 min	[93-116] alpha crystallin B	K103
635.9282	5	3174.6019	3174.6058	21 min	[91-116] alpha crystallin B	K92, K103
668.3391	5	3336.6564	3336.6586	21 min	[91-116] alpha crystallin B*	K92, K103
737.8664	4	2947.4343	2947.4424	21 min	[93-116] alpha crystallin B	K103
691.3524	6	4142.0674	4142.0821	25 min	[83-116] alpha crystallin B	K90, K92, K103
631.3107	5	3151.5144	3151.5211	26 min	[79-103] alpha crystallin A	K88, K99
704.8385	6	4222.9840	4222.9944	26 min	[79-112] alpha crystallin A	K88, K99
788.8867	4	3151.5155	3151.5211	26 min	[79-103] alpha crystallin A	K88, K99
845.6072	5	4222.9969	4222.9944	26 min	[79-112] alpha crystallin A	K88, K99
878.0158	5	4385.0399	4385.0473	26 min	[79-112] alpha crystallin A*	K88, K99
1097.2668	4	4385.0359	4385.0473	26 min	[79-112] alpha crystallin A*	K88, K99
581.9777	3	1742.9096	1742.9091	27 min	[66-78] alpha crystallin A	K70
663.7227	5	3313.5744	3313.5739	27 min	[79-103] alpha crystallin A*	K88, K99
894.4367	5	4467.1444	4467.1435	28 min	[117-157] alpha crystallin A	K145
1308.6389	2	2615.2621	2615.2755	28 min	[79-99] alpha crystallin A	K88
725.1298	4	2896.4879	2896.4858	29 min	[66-88] alpha crystallin A	K70, K78
998.9775	4	3991.8787	3991.8779	29 min	[120-157] alpha crystallin A	K145
725.8888	4	2899.5239	2899.5179	30 min	[55-78] alpha crystallin A	K70
801.6782	4	3202.6815	3202.6721	30 min	[121-149] alpha crystallin B	K121

697.3621	6	4178.1256	4178.1212	31 min	[66-99] alpha crystallin A	K70, K78, K88
724.3716	6	4340.1826	4340.1740	31 min	[66-99] alpha crystallin A*	K70, K78, K88
801.6767	4	3202.6755	3202.6721	31 min	[121-149] alpha crystallin B	K121
1086.0512	4	4340.1735	4340.1740	31 min	[66-99] alpha crystallin A*	K70, K78, K88
1247.3070	3	3738.8975	3738.8920	32 min	[71-99] alpha crystallin A*	K78, K88

* Asterisk indicates diglycated peptides. Retention time range used for averaging mass spectra [min]. Details of LC-MS are described in Materials and Methods

Table S7 The glycated fragments identified in experiment A8 (Materials and Methods). AA-potential glycated lysine residues

m/z	z	exp. mass	calc. mass	time	sequence	AA
651.8473	2	1301.6789	1301.6715	17 min	[164-174] alpha crystallin B	K166
704.7381	3	2111.1908	2111.1838	19 min	[158-175] alpha crystallin B	K166, K174
1056.6002	2	2111.1847	2111.1838	19 min	[158-175] alpha crystallin B	K166, K174
614.8941	5	3069.4314	3069.4177	20 min	[89-112] alpha crystallin A	K99
662.0391	3	1983.0938	1983.0888	20 min	[158-174] alpha crystallin B	K166
768.3648	4	3069.4279	3069.4177	20 min	[89-112] alpha crystallin A	K99
992.5535	2	1983.0913	1983.0888	20 min	[158-174] alpha crystallin B	K166
635.9294	5	3174.6079	3174.6058	21 min	[91-116] alpha crystallin B	K92, K103
794.6584	4	3174.6023	3174.6058	21 min	[91-116] alpha crystallin B	K92, K103
983.4875	3	2947.4390	2947.4424	21 min	[93-116] alpha crystallin B	K103
704.3527	5	3516.7244	3516.7134	22 min	[93-120] alpha crystallin B	K103
749.7863	5	3743.8924	3743.8768	22 min	[91-120] alpha crystallin B	K92, K103
697.2106	7	4873.4194	4873.4060	25 min	[83-120] alpha crystallin B*	K90, K92, K103
1219.3636	4	4873.4231	4873.4060	25 min	[83-120] alpha crystallin B*	K90, K92, K103
581.9828	3	1742.9249	1742.9091	27 min	[66-78] alpha crystallin A	K70
1051.5202	3	3151.5371	3151.5211	27 min	[79-103] alpha crystallin A	K88, K99
1262.1521	4	5044.5771	5044.5683	30 min	[75-116] alpha crystallin B	K82, K90, K92,K103

* Asterisk indicates diglycated peptides. Retention time range used for averaging mass spectra [min]. Details of LC-MS are described in Materials and Methods

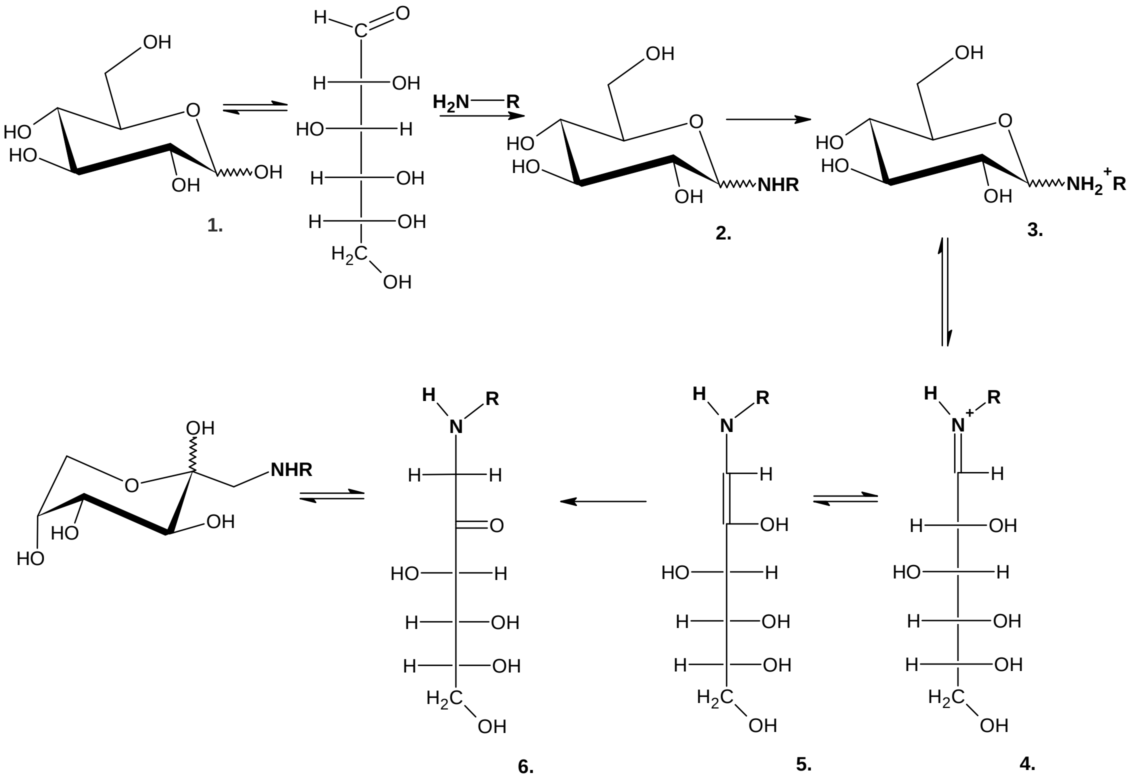

Fig. S1 The scheme of Amadori rearrangement

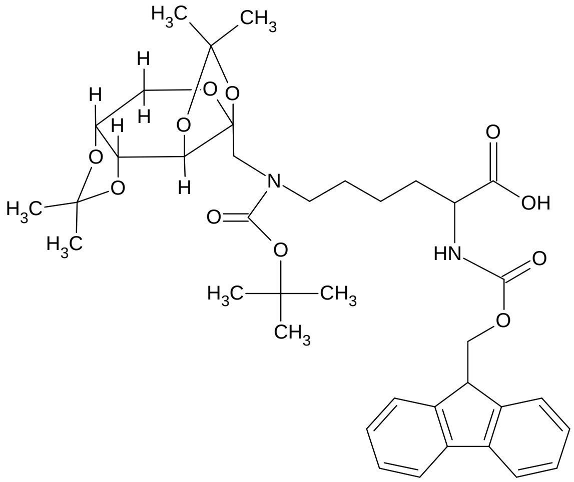

Fig. S2 The structure of Fmoc-Lys(*i*,*i*-Fru,Boc)-OH

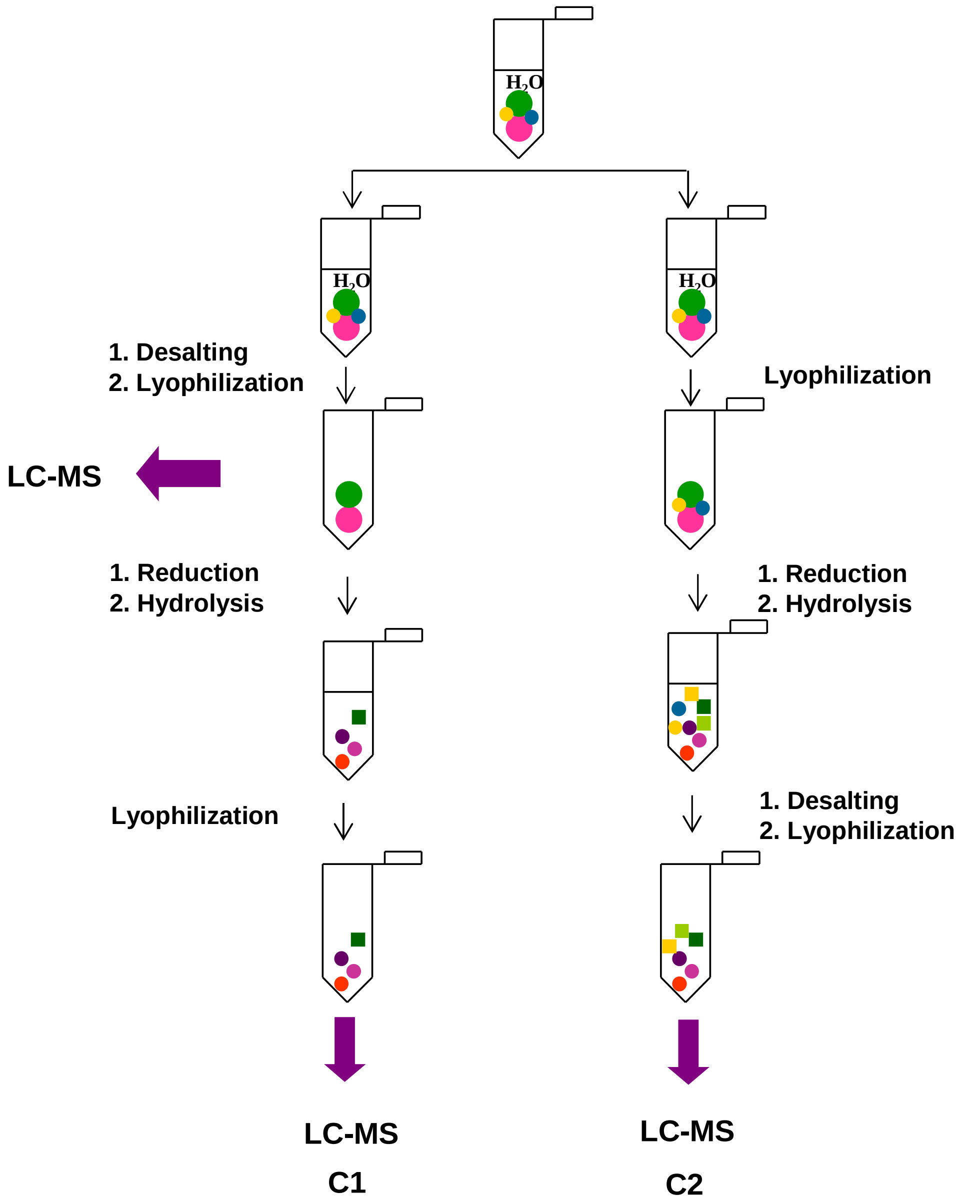

Fig. S3 Diagram of the procedures for preparation of the biological material for LC-MS analysis (C1-C2)

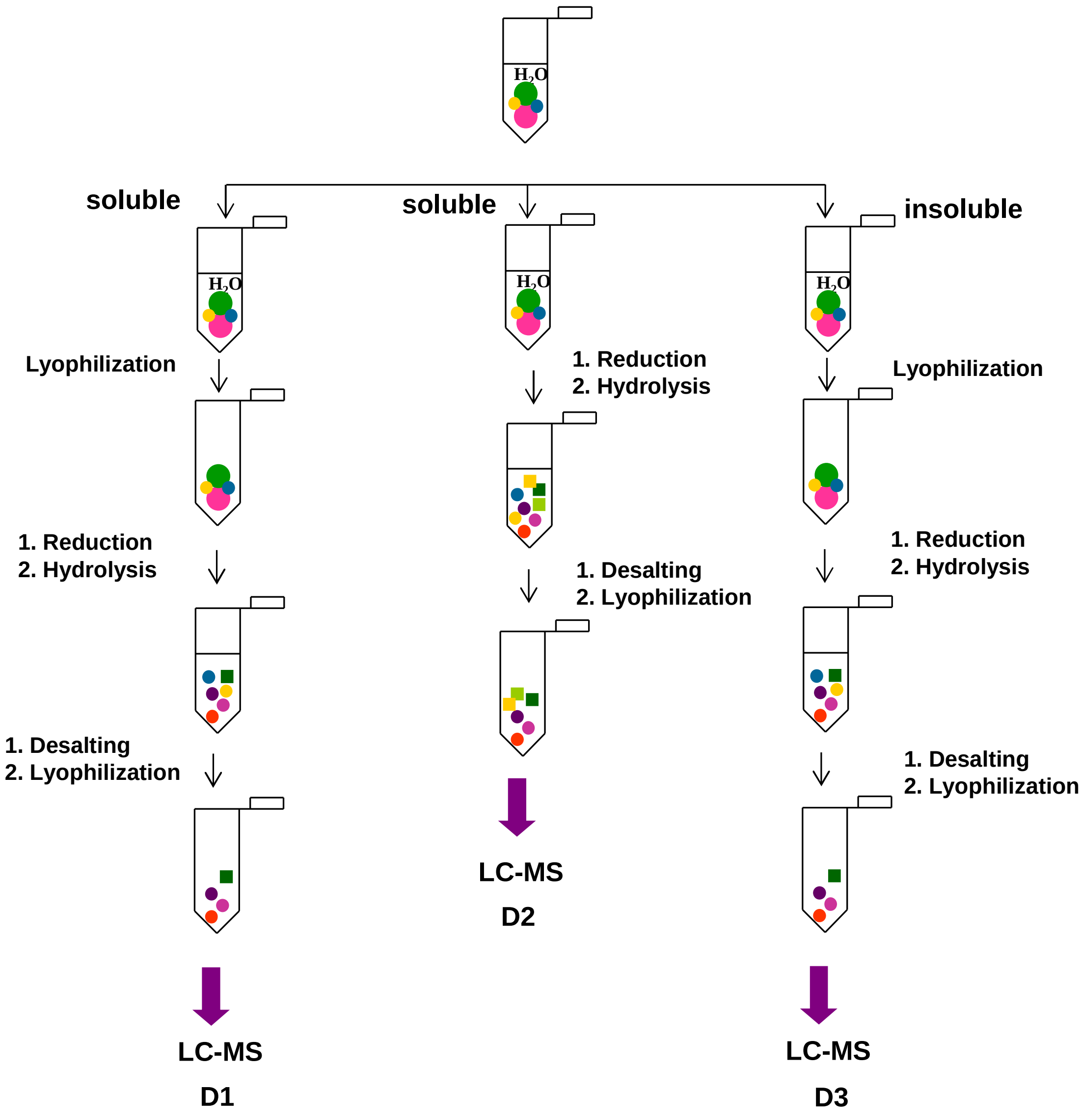

Fig.S4 Diagram of the procedures for preparation of the biological material for LC-MS analysis (D1-D3)

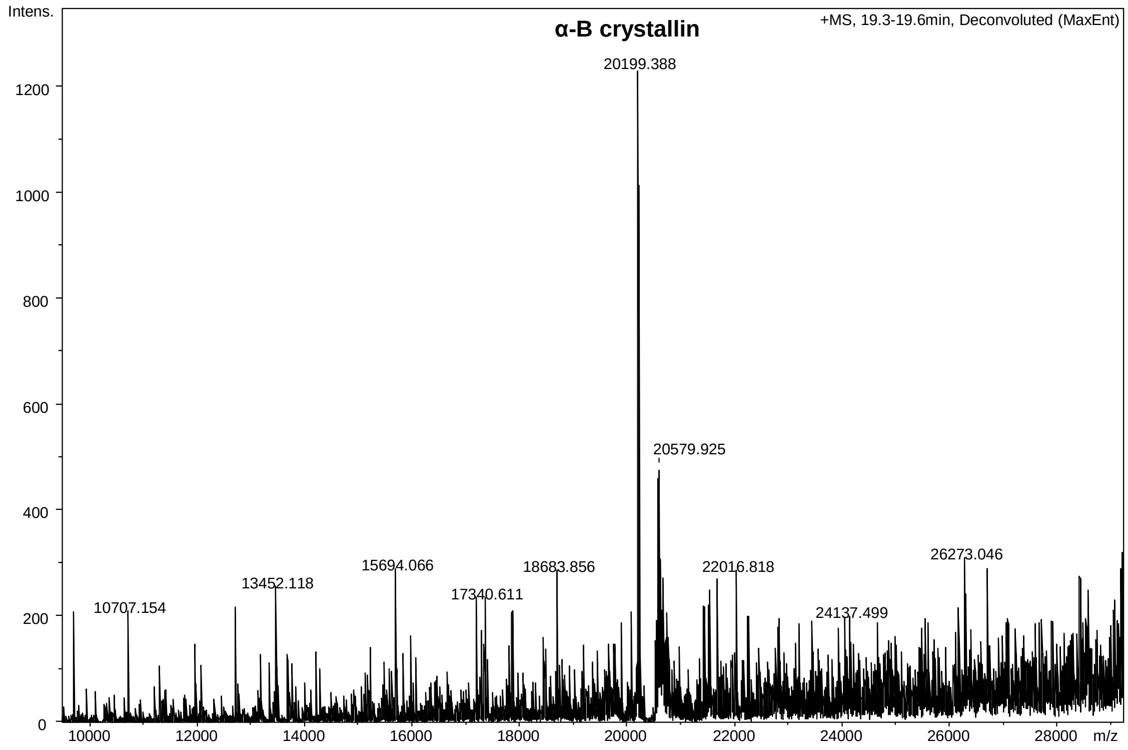

Fig. S5 Deconvoluted ESI-MS spectrum of α-B crystallin (sample C1)

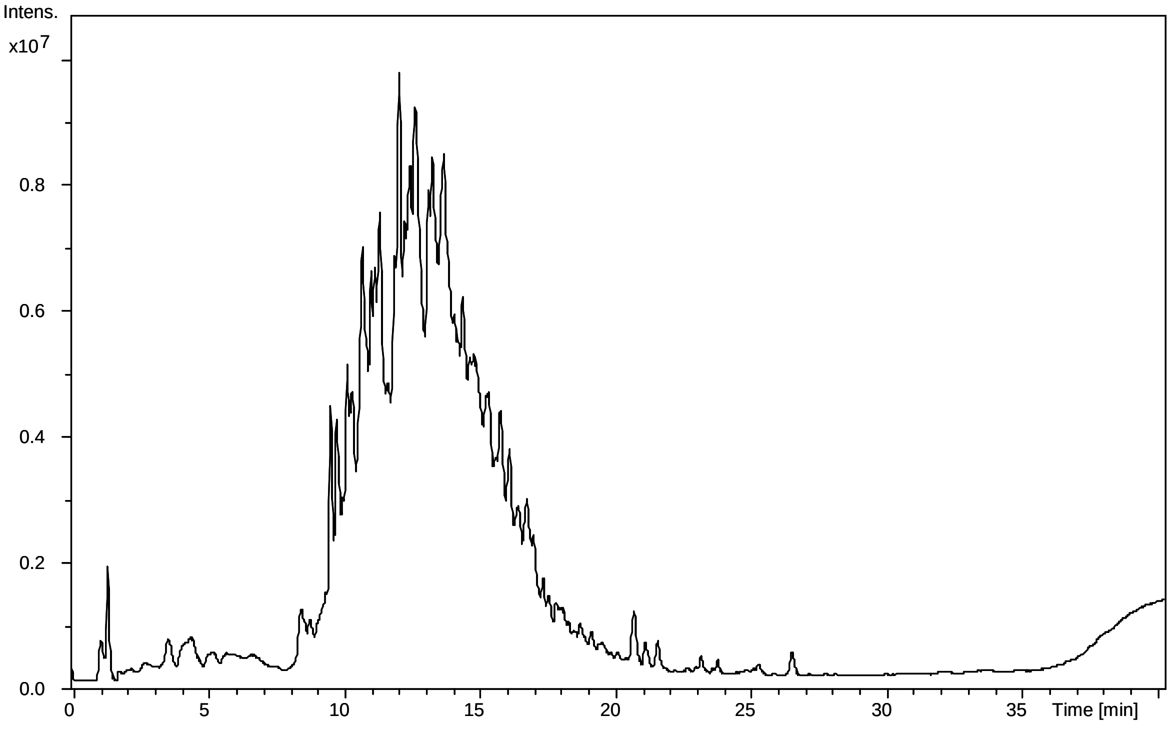

Fig. S6 The representative chromatogram for sample C2

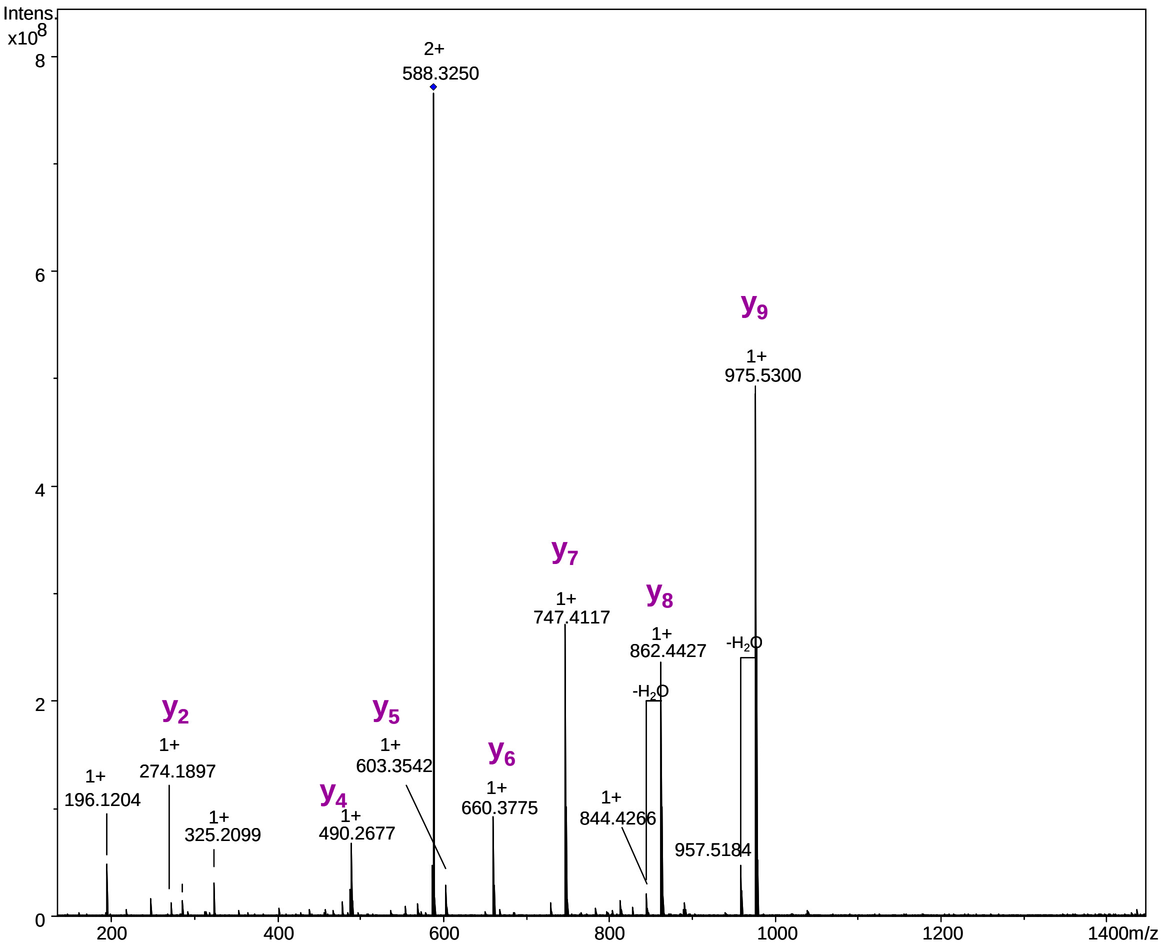

Fig. S7 MS/MS spectrum for TVLDSGISEVR [55-65] fragment of α-A crystallin (parent ion 588.32 m/z; collision energy 15eV)

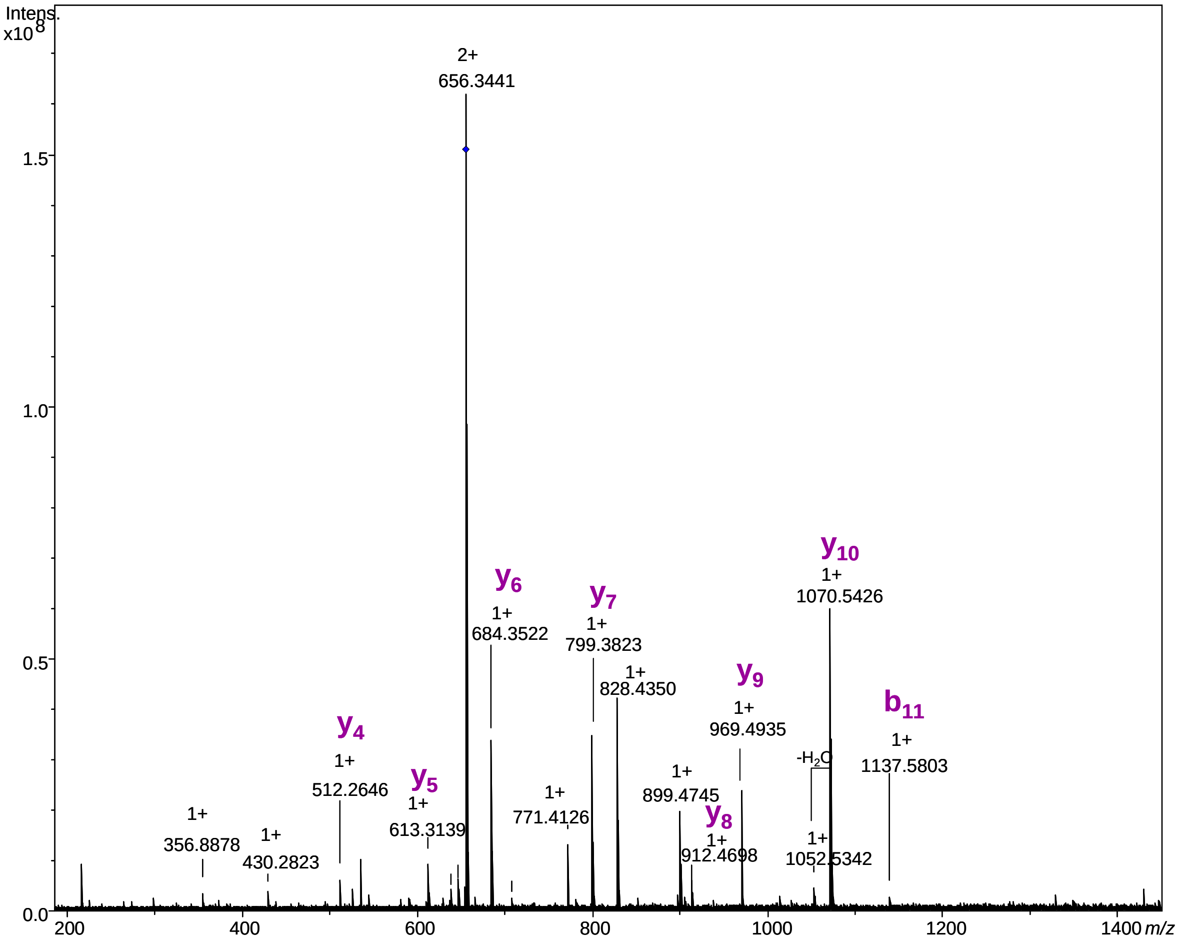

Fig. S8 MS/MS spectrum for IQTGLDATHAER [146-157] fragment of αA- crystallin (parent ion 656.34 m/z; collision energy 15eV)

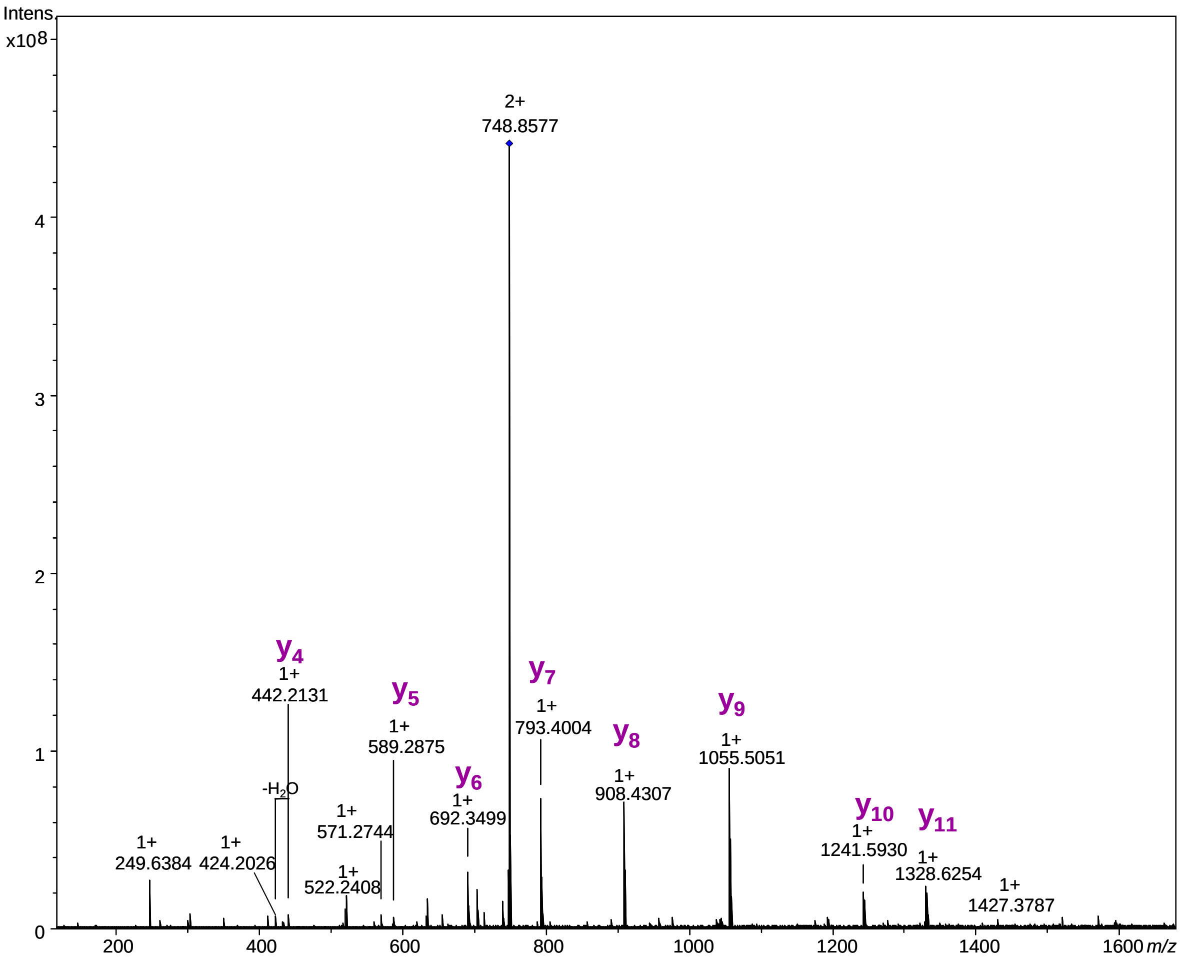

Fig. S9 MS/MS spectrum for APSWFDTGLSEMR [57-69] fragment of αB- crystallin (parent ion 748.86 m/z; collision energy 15eV)
